# The Role of Sarcopenia and Body Composition in Lung Transplant Candidates: A Review

**DOI:** 10.7759/cureus.92438

**Published:** 2025-09-16

**Authors:** Aman Aher, Arya Kermanshah, Samal Badhuli, Sarv Priya, Sameer Samtani, Pritish Aher

**Affiliations:** 1 Nutrition and Exercise Physiology, University of Missouri, Columbia, USA; 2 Anesthesiology, University of Miami Miller School of Medicine, Miami, USA; 3 Radiology, University of Miami, Miami, USA; 4 Radiology, University of Iowa Hospitals and Clinics, Iowa City, USA; 5 Radiology, University of Miami Health System, Jackson Memorial Hospital, Miami, USA; 6 Radiology, University of Miami Miller School of Medicine, Miami, USA

**Keywords:** body composition indices, ct based delineation, lung and cardiac transplantation, musculo-skeletal health, : sarcopenia

## Abstract

CT-derived body composition metrics such as skeletal muscle index (SMI), pectoralis muscle index (PMI), muscle radiation attenuation (MRA), and visceral fat area (VFA) have emerged as promising prognostic tools in lung transplant candidates. This systematic review evaluates their utility in pre- and post-transplant risk assessment and rehabilitation through a detailed literature search of PubMed, Google Scholar, and Excerpta Medica Database (EMBASE), identifying 21 relevant studies. Reduced muscular area (SMI) and quality (MRA) were associated with increased mortality, prolonged mechanical ventilation, and extended ICU stay, while low MRA correlated with higher rates of infection and graft dysfunction. Elevated VFA was linked to increased metabolic complications, and composite phenotypes such as sarcopenic obesity conferred additional risk. These findings suggest that CT-derived body composition metrics provide valuable, objective insights for transplant risk stratification and recovery. Standardized imaging protocols and integration with functional assessments are needed to support their widespread clinical adoption and optimize outcomes for high-risk lung transplant candidates.

## Introduction and background

Sarcopenia, frailty, and cachexia are interrelated conditions that profoundly impact outcomes in lung transplantation. Sarcopenia, the specific loss of skeletal muscle mass and strength, is a core physical component of the broader syndrome of frailty, which describes a state of reduced physiological reserve and increased vulnerability to stressors. In patients with end-stage lung disease, cachexia, a metabolic wasting syndrome driven by systemic inflammation and underlying illness, is a primary driver of both sarcopenia and frailty. This triad frequently coexists and creates a high-risk phenotype for lung transplant candidates. The presence of these conditions predicts increased mortality on the waitlist due to depleted energy reserves and an inability to withstand the catabolic burden of chronic disease. Furthermore, they are strongly associated with post-transplant complications, including prolonged mechanical ventilation, higher rates of infection and rejection, extended hospital stays, and reduced overall survival. Consequently, the assessment of sarcopenia, frailty, and cachexia is now integral to pre-transplant evaluation, serving as crucial biomarkers for risk stratification and potential targets for pre-habilitation strategies aimed at optimizing patients for surgery [1].

Diagnostically, sarcopenia is defined by gait speed <1 m/s and appendicular lean mass index (ALMI) ≤7.23 kg/m² (men) or ≤5.67 kg/m² (women) [2]. Screening tools include the strength, assistance with walking, rise from a chair, climb stairs, and falls (SARC-F) questionnaire (score ≥4 indicating probable sarcopenia) and functional assessments such as grip strength (<27 kg for men, <16 kg for women) and five-repetition chair stand (>15 sec) [3-7]. Reduced grip strength specifically predicts prolonged hospitalization and mortality. The European Working Group on Sarcopenia in Older People (EWGSOP)2 recommends additional assessments, including gait speed, short physical performance battery (SPPB), and timed up and go (TUG) tests, for comprehensive evaluation [8]. 

While MRI remains the gold standard for muscle quantification, CT and dual-energy X-ray absorptiometry (DXA) offer practical alternatives [9]. CT-derived measures, including skeletal muscle index (SMI), muscle radiation attenuation (MRA), and pectoralis muscle index (PMI), provide prognostic value, with low PMI associated with poorer transplant outcomes. Visceral fat area (VFA) correlates with systemic inflammation and muscle dysfunction, while subcutaneous fat may have neutral or protective metabolic effects [1]. 

## Review

Methodology

Literature Review Strategy

**Figure 1:**
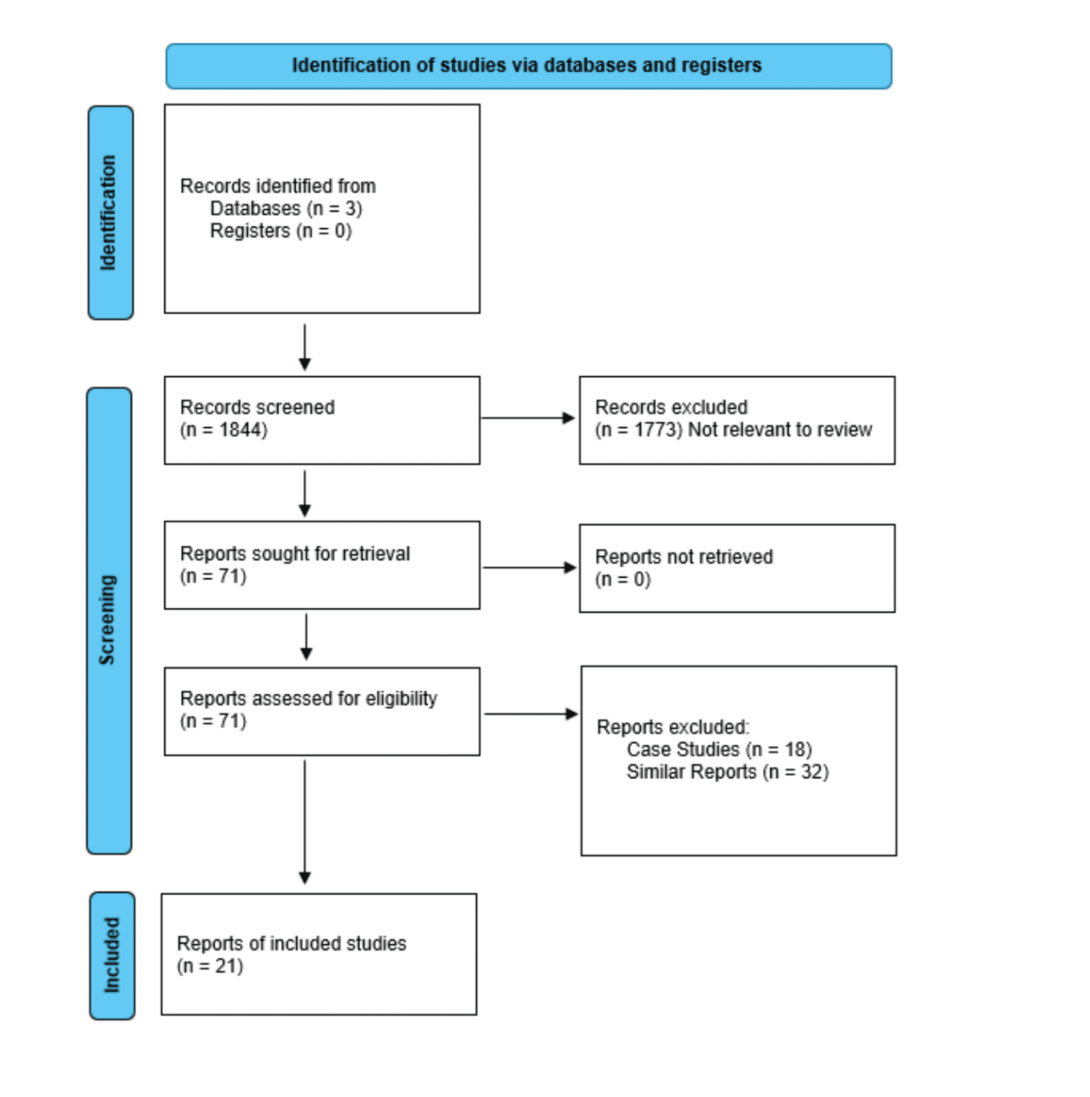
PRISMA Consort Flow

A systematic literature review was performed using the widely accepted PRISMA (Figure 1) guidelines for articles published between January 2000 and March 2025. The total number of articles found: PubMed was 872, Embase was 579, and Google Scholar was 393. The search plan consisted of using appropriate keywords along with the Boolean operators like “AND” as well as “OR”, depending on the nature of the search. The primary keywords used were ("lung transplant" OR "lung transplantation") AND sarcopenia AND (radiology OR "computed tomography" OR “CT”).

Eligible studies involved adult participants (≥18 years) with chronic lung disease affecting the parenchyma, airways, or pulmonary vasculature who were undergoing evaluation for lung transplantation. Only studies reporting thoracic CT imaging data with at least one post-transplant clinical outcome were considered, encompassing both prospective and retrospective cohort studies as well as randomized and non-randomized controlled trials. To ensure consistency, the review was limited to articles published in English. The methodological quality and risk of bias of included studies were assessed using the Newcastle-Ottawa Scale for cohort studies and the Cochrane risk of bias tool for randomized controlled trials, evaluating factors such as selection bias, outcome measurement, and statistical adjustment for confounders. 

Results

The included studies demonstrated that CT-derived body composition parameters provide valuable prognostic information in lung transplantation (Table 1). Skeletal muscle index (SMI), measured at thoracic or lumbar levels, showed significant associations with post-transplant outcomes, including mortality, duration of mechanical ventilation, and ICU length of stay. PMI emerged as a thoracic-specific predictor of survival and graft dysfunction risk. 

**Table 1:** Definitions and Clinical Significance SMI: skeletal muscle index, PMI - pectoralis muscle index, VFA - visceral fat area, SFA - subcutaneous fat area. CSA: cross-sectional area.

Metric	Full Name	Calculation / Measurement	Clinical Relevance / Significance
SMI	Skeletal Muscle Index	CSA of skeletal muscles (e.g., pectoralis, paraspinal) at a standardized level (e.g., T4, L3), normalized to height².	A diagnostic criterion for sarcopenia. Associated with increased mortality and complications in lung transplant candidates.
PMI	Pectoralis Muscle Index	CSA of the pectoralis muscles at the aortic arch (T4), normalized to height².	Particularly relevant in thoracic surgery and lung transplantation, as it is accessible on routine chest CT scans.
Muscle Attenuation	(Measured in Hounsfield Units - HU)	Quantifies fat infiltration (myosteatosis) in muscle tissue.	Lower values (<30 HU) indicate poor muscle quality and are associated with worse clinical outcomes.
VFA	Visceral Fat Area	CSA of visceral fat at the L3 vertebra or aortic arch.	High VFA is linked to metabolic syndrome, inflammation, and increased surgical risk.
SFA	Subcutaneous Fat Area	CSA of subcutaneous fat at the same axial level (e.g., L3 or T4).	Provides context for overall adiposity; less strongly associated with metabolic risk than VFA.

Muscle radiation attenuation (MRA) served as an important indicator of muscle quality, with lower values correlating with increased infection rates and graft dysfunction. Visceral fat area (VFA) was consistently linked to metabolic complications, while subcutaneous fat area (SFA) showed weaker associations with postoperative outcomes. The combination of low muscle mass and high adiposity (sarcopenic obesity) appeared to confer particularly poor outcomes in transplant recipients [10-12]. These findings highlight the clinical relevance of CT-based body composition analysis in lung transplantation, though variations in measurement methodologies between studies suggest a need for standardization in future research and clinical application. 

Discussion

Sarcopenia, as defined by the EWGSOP, is a syndrome marked by progressive loss of skeletal muscle mass and strength, leading to adverse outcomes such as disability, poor quality of life, and increased mortality.  Sarcopenia is classified into primary and secondary forms. Primary sarcopenia results solely from aging, while secondary sarcopenia is driven by identifiable causes such as chronic diseases, malnutrition, metabolic dysfunction, or physical inactivity. This distinction is crucial in clinical settings, particularly transplant care, where primary sarcopenia may require long-term monitoring, and secondary sarcopenia often benefits from targeted interventions like nutritional support and prehabilitation. 

The progression of sarcopenia occurs in three stages: presarcopenia, sarcopenia, and severe sarcopenia. Presarcopenia is characterized by low muscle mass alone, detectable only through precise imaging. Sarcopenia involves reduced muscle mass combined with either decreased strength or impaired physical function, while severe sarcopenia includes all three deficits. This staging helps tailor interventions, from early nutritional strategies in presarcopenia to intensive rehabilitation in advanced cases. Sarcopenia often coexists with frailty, a condition of reduced physiological resilience, and cachexia, a metabolic syndrome involving muscle loss due to underlying illness. Both conditions amplify risks such as falls, hospitalization, and mortality. 

Diagnosis relies on criteria such as slow gait speed and low appendicular lean mass index, with screening tools like the SARC-F questionnaire and functional tests aiding early detection. Reduced grip strength, for example, is linked to worse clinical outcomes. Advanced imaging techniques, including CT and MRI, provide objective measures of muscle mass and quality, with CT-derived indices offering prognostic value in transplant patients. Additionally, visceral fat accumulation is associated with inflammation and muscle dysfunction, while subcutaneous fat may have a neutral or protective effect. These insights guide personalized management strategies to mitigate sarcopenia’s impact on health and recovery. 

Skeletal Muscle Metrics and Clinical Impact

Skeletal muscle index (SMI) emerged as a particularly robust predictor, with established thresholds (<7.0 kg/m² for men and <5.7 kg/m² for women) demonstrating strong associations with functional impairment [13]. SMI is calculated as the cross-sectional area (CSA) of skeletal muscles (e.g., pectoralis, paraspinal) at a standardized axial level (e.g., T4 or L3), normalized to height². Low SMI is a diagnostic criterion for sarcopenia and has been associated with increased mortality and complications in lung transplant candidates. Multiple studies confirmed that these SMI cutoffs correlate significantly with reduced six-minute walk distance (6MWD), diminished pulmonary capacity, and decreased physical activity levels in transplant candidates. The classification system proposed by Janssen et al., which categorizes sarcopenia into Class I and II based on SMI deviations from population means, proved clinically relevant in the transplant population. Class II sarcopenia, representing more severe muscle loss, was associated with substantially higher risks of disability and poor outcomes [13-16]. Longitudinal data from Nikkuni et al. offered encouraging findings regarding post-transplant recovery, showing that more than half of sarcopenic patients achieved non-sarcopenic status within one year after transplantation, with significant improvements in erector spinae muscle cross-sectional area [17]. 

The prognostic significance of muscle mass extends beyond global measures like SMI. Studies focusing on specific muscle groups yielded important insights. Kifjak et al. and Weig et al. identified lumbar and paraspinal muscle depletion as particularly strong predictors of adverse outcomes, including prolonged ICU stays, extended mechanical ventilation requirements, and poorer rehabilitation outcomes [17]. Notably, these associations remained significant even after adjusting for body mass index (BMI), suggesting that muscle mass provides independent prognostic information beyond traditional nutritional status indicators. 

Alternate Muscle and Fat Indicators

The review highlighted the complementary value of muscle quality assessment through metrics like muscle radiodensity attenuation (MRA). Cho et al. established that low muscle attenuation (MHI <28.07 cm²/m²) serves as an independent risk factor for increased mortality and longer ICU duration [15-19]. The studies consistently demonstrated that MRA values decline with both advancing age and increasing BMI, with these changes explaining a substantial proportion of variance in overall muscle quality. 

Regional muscle measures, particularly the PMI, provided unique insights. PMI focuses on the CSA of the pectoralis muscles at the level of the aortic arch (T4), normalized to height². PMI is relevant in thoracic surgery and lung transplantation, as it reflects the muscle mass most accessible on routine chest CT scans. While Hu et al. found that PMI did not predict transplant likelihood, it showed significant associations with shorter hospital stays and better recovery trajectories [19]. This finding suggests that thoracic-specific muscle measures may be particularly relevant for postoperative recovery, though less influential in pre-transplant risk assessment. 

The analysis of adiposity metrics revealed complex relationships with transplant outcomes. Visceral fat area (VFA) demonstrated particularly strong associations with metabolic complications, consistent with its known systemic effects. VFA measures the CSA of visceral fat at the level of the L3 vertebra or aortic arch. However, the relationship between VFA and outcomes was not linear, with Al-Naamani et al. reporting reduced pulmonary hypertension risk at moderate VFA levels, and Anderson et al. documenting a U-shaped relationship between VFA and frailty. Subcutaneous fat area (SFA) showed generally weaker associations with outcomes, though some studies suggested potential protective effects in certain contexts. The most striking findings emerged from analyses of body composition phenotypes. Kifjak et al. demonstrated that the "low muscle, high fat" or sarcopenic obesity phenotype conferred particularly poor prognosis, associated with worse pre-transplant status, higher Lung Allocation Scores (LAS), and greater need for ECMO support [17]. 

The review uncovered important temporal patterns in body composition changes and their clinical implications. Post-transplant recovery of muscle mass emerged as a common finding, with Nikkuni et al. documenting significant improvements in muscle parameters within the first year after transplantation [17]. However, the rate and extent of recovery varied substantially between patients, suggesting potential opportunities for targeted rehabilitation interventions. 

MRA and PMI measurements proved valuable not only for pre-transplant risk assessment but also for monitoring post-transplant recovery. Several studies linked improvements in these metrics to shorter recovery times and better functional outcomes [19], supporting their use in tracking rehabilitation progress. 

Mortality and long-term outcomes

Despite the strong associations between muscle/fat measures and perioperative outcomes, their link to long-term survival remains inconclusive. Post-transplant sarcopenic status was not significantly tied to mortality, nor were measures like PMI or MRA predictive of waitlist death or transplant likelihood [16-21]. Instead, traditional factors like transplant type, acute rejection episodes, and pre-transplant FEV₁ emerged as stronger predictors of long-term survival. These findings emphasize that while sarcopenia and body composition affect short-term clinical trajectories, long-term survival hinges more on respiratory function and procedural factors than on baseline muscle or fat mass.

Standardization and Future Needs

The review [22] identified significant methodological challenges, particularly regarding the standardization of measurement protocols. Variability in anatomical reference points (e.g., T4 vs. L3), analysis software, and diagnostic thresholds limited cross-study comparability and highlighted the need for consensus guidelines. 

Future research in this field should focus on several key priorities. First, prospective longitudinal studies are needed to establish causal relationships between body composition changes and transplant outcomes, as current evidence primarily comes from retrospective analyses. Second, the development of standardized protocols for image acquisition and analysis is crucial to address current variability in measurement techniques across institutions. Third, researchers should work toward integrating CT-based metrics with functional assessments like grip strength and gait speed to create more comprehensive evaluation tools. Fourth, the development of multimodal assessment frameworks that combine imaging, functional, and clinical data could significantly improve risk prediction. Finally, investigations into targeted interventions based on specific body composition profiles may lead to more personalized and effective prehabilitation strategies. 

These findings have important clinical implications for lung transplant practice. The evidence supports using combined muscle and fat metrics for improved pre-transplant risk stratification, allowing clinicians to better identify high-risk candidates. This approach also facilitates the selection of patients who may benefit most from prehabilitation programs. Post-transplant monitoring of body composition changes can provide valuable insights into recovery progress and help guide rehabilitation efforts. Perhaps most importantly, these metrics enable more personalized rehabilitation planning by identifying specific deficits in muscle mass or quality that need to be addressed. Together, these applications have the potential to significantly improve outcomes throughout the transplant process, from initial evaluation through long-term recovery. 

## Conclusions

This review highlights the value of CT-derived body composition indices in lung transplant candidates. These indices offer predictive insights into post-transplant mortality and complications, highlighting the potential for early intervention and personalized rehabilitation strategies to improve patient outcomes and enhance recovery. Specifically, low SMI and MRA are expected to correlate with higher rates of perioperative complications and mortality, independent of BMI, while elevated VFA and sarcopenic obesity may amplify post-transplant metabolic and infectious risks. The review further proposes that integrating these imaging-based metrics with functional assessments like the 6-minute walk distance (6MWD) and grip strength could enhance risk stratification and inform tailored rehabilitation strategies, although their influence on long-term survival may be limited by procedural and immunologic determinants.
